# Systemic Immune Activation Profiles of HIV-1 Subtype C-Infected Children and Their Mothers

**DOI:** 10.1155/2016/9026573

**Published:** 2016-02-25

**Authors:** Tinyiko G. Makhubele, Helen C. Steel, Ronald Anderson, Gisela van Dyk, Annette J. Theron, Theresa M. Rossouw

**Affiliations:** ^1^Institute for Cellular and Molecular Medicine, Department of Immunology, Faculty of Health Sciences, University of Pretoria, Pretoria 0001, South Africa; ^2^Tshwane Academic Division of the National Health Laboratory Service, Pretoria, South Africa; ^3^Department of Family Medicine, Faculty of Health Sciences, University of Pretoria, Pretoria, South Africa

## Abstract

Little is known about immune activation profiles of children infected with HIV-1 subtype C. The current study compared levels of selected circulating biomarkers of immune activation in HIV-1 subtype C-infected untreated mothers and their children with those of healthy controls. Multiplex bead array, ELISA, and immunonephelometric procedures were used to measure soluble CD14 (sCD14), beta-2 microglobulin (*β*2M), CRP, MIG, IP-10, and transforming growth factor beta 1 (TGF-*β*1). Levels of all 6 biomarkers were significantly elevated in the HIV-infected mothers and, with the exception of MIG, in their children (*P* < 0.01–*P* < 0.0001). The effects of antiretroviral therapy (ART) and maternal smoking on these biomarkers were also assessed. With the exception of TGF-*β*1, which was unchanged in the children 12 months after therapy, initiation of ART was accompanied by decreases in the other biomarkers. Regression analysis revealed that although most biomarkers were apparently unaffected by smoking, exposure of children to maternal smoking was associated with a significant increase in IP-10. These findings demonstrate that biomarkers of immune activation are elevated in HIV-infected children pre-ART and decline, with the exception of TGF-*β*1, after therapy. Although preliminary, elevation of IP-10 in smoke-exposed infants is consistent with a higher level of immune activation in this group.

## 1. Introduction

Persistent immune activation and inflammation, even in the setting of highly active antiretroviral therapy (HAART) mediated viral suppression, are an emerging feature of chronic human immunodeficiency virus (HIV) infection and are considered to be the driving force underpinning CD4^+^ T cell depletion and progression to acquired immunodeficiency syndrome (AIDS) [1]. Markers of chronic immune activation in HIV-infected adults include enhanced expression of activation markers on peripheral blood T cells, B cells, monocytes, dendritic cells, and natural killer cells [2–4] and high levels of circulating beta-2 microglobulin (*β*2M) and soluble CD14 (sCD14), as well as various proinflammatory and anti-inflammatory cytokines and chemokines such as tumor necrosis factor alpha (TNF*α*), interleukin 6 (IL-6), IL-1*β*, transforming growth factor beta 1 (TGF-*β*1), MIG, IP-10, MIP-1*α*, MIP-1*β*, and RANTES [2, 4, 5]. However, little is known about the immune activation profiles of children infected with HIV-1 subtype C.

Long-term complications, possibly as a consequence of chronic immune activation, are emerging as the greatest challenges facing HIV-infected individuals [6, 7]. In children, these complications include atherosclerotic cardiovascular disease (CVD) and CVD risk factors such as dyslipidemia, insulin resistance, and obesity [6, 8–10]. Chronic immune activation may also play a role in HIV-related complications such as malignancies and neurological diseases [11, 12].

Like HIV infection, cigarette smoking is also associated with a chronic, albeit low-grade, systemic inflammatory response [13], possibly exacerbating chronic immune activation and the associated risks in HIV-infected smokers. In this context, it is noteworthy that as many as 50–70% of those infected with HIV are current smokers [14], although the prevalence is reported to be much lower in African populations, especially women. In addition to increasing the risk for development of life-threatening respiratory infections, smoking is also associated with a significantly increased prevalence of non-AIDS-defining illnesses [15]. Given the well-recognized association between parental smoking and an increased risk of lower respiratory infection in infancy and early childhood [16, 17], it is worrying, but unproven, that this risk may be even higher in HIV-infected children.

The primary objective of this study was to investigate systemic immune activation profiles of children and their mothers infected with HIV subtype C. Secondary objectives included evaluation of the effects of both HAART and exposure to maternal smoking on systemic immune activation profiles in children. The panel of biomarkers was based on the findings of recent studies and included those which are either responsive (*β*2M, IP-10, and MIG) or unresponsive to HAART (sCD14, TGF-*β*1), the latter group consistent with chronic immune activation [4, 5].

## 2. Methods

### 2.1. Study Population

HIV-infected mothers and their infants presenting for care at the Paediatric Immunology Clinic at Kalafong Provincial Tertiary Hospital in Pretoria, South Africa, after failed prevention of mother-to-child transmission (PMTCT) were included in this study. Ethics approval was granted by the Research Ethics Committee of the Faculty of Health Sciences at the University of Pretoria (Ethics Committee Approval Numbers: 159/2009 and 175/2013). All mothers gave informed consent on behalf of themselves and their infants.

Eighty-six mother-infant pairs were recruited at the time infants presented to the clinic for initiation of HAART and followed prospectively for up to 24 months as part of a HIV drug resistance study. Of these, 46 mother-infant pairs had adequate preinitiation plasma samples for inclusion in this study (named the mother-infant group). Twenty-two infants (including 16 from the mother-infant group) had adequate follow-up samples around month 7 (median 7.45; IQR 7.1–7.8 months) and month 13 (median 12.85; IQR 12.2–13.5 months) after initiation (named the longitudinal group). Children were evaluated for the presence of virological suppression, defined as HIV viral load below 25 RNA copies/mL plasma. Twenty healthy, nonsmoking HIV-uninfected females were included to serve as controls for the mothers. Twenty-nine healthy, HIV-exposed, but uninfected, paediatric controls were recruited from the 6-week HIV PCR programme and residual blood used. Controls closer in age to the study infants could not be accessed since blood is not routinely collected in healthy children of this age group. Mothers of the HIV-infected infants and their controls, as well as the pediatric controls, were tested only once at the outset.

Whole blood samples were collected in EDTA vacutainers, processed within 24 hours to separate the plasma component by centrifugation, and stored at −70°C for up to 24 months. CD4^+^ T-lymphocyte counts (CD4^+^), CD8^+^ T-lymphocyte counts (CD8^+^) (Beckman Coulter SA (Pty) Ltd.), and HIV-1 RNA (VL) (Nuclisens HIV-1 Viral Load Assay v1.2 or v2.0) were measured by standard flow cytometric and PCR-based procedures, respectively, according to the manufacturer's instructions. Mothers and infants were routinely screened for* Mycobacterium tuberculosis* and for other opportunistic infections as clinically indicated. Routine screening for other viral infections was not done.

### 2.2. Measurement of Circulating Biomarkers of Immune Activation and Cotinine

Biomarkers were selected on the basis of previous studies: *β*2M, IP-10, and MIG have been shown to decrease in HIV-infected patients virologically suppressed on HAART, while sCD14 and TGF-*β*1 remain elevated, suggesting chronic immune activation [4, 5]. IP-10, MIG, and TGF-*β*1 were measured using the multiplex bead array system (Bio-Rad Laboratories Inc., Hercules, CA, USA), the lower limits of detection of these chemokines/cytokine being 3.5, 0.26, and 1.83 picograms (pg)/mL, respectively, according to the manufacturer. sCD14 was tested using a conventional sandwich ELISA procedure (Abcam, Cambridge, MA, USA), while high sensitivity C-reactive protein (hsCRP) and *β*2M were assayed by nephelometry (Siemens Healthcare Diagnostics, South Africa). Cotinine as an objective measure of cigarette smoke exposure was measured using an ELISA procedure (Calbiotech, Spring Valley, CA, USA), with levels above 14 nanograms (ng)/mL taken as being positive for active smoking, as recommended by the Society for Research on Nicotine and Tobacco Biochemical Assessment Working Group [18]. Details of smoking history (type and duration of smoking exposure) had not been collected.

### 2.3. Data Analysis and Statistics

All data followed a nonnormal distribution and are presented as the median, minimum, and maximum concentrations (range) for each of the measured cytokines/chemokines, as well as for cotinine. Median concentrations of each parameter were compared between and within cohorts using the Mann-Whitney test for independent groups (Tables 1 and 2) and the Wilcoxon matched paired test (Table 3) for the longitudinal group. Multiple linear regression analysis was performed to assess the relationship between the cytokines and various predictor variables. Statistical significance as calculated by the InStat or Prism software statistics programmes (GraphPad, La Jolla, CA) was set at *P* ≤ 0.05.

## 3. Results

### 3.1. Demographic Data of Paired Mothers and Children at Baseline

The median age of the mothers was 27 years (range 20–44) and that of the controls was 26 years (range 23.7–32.5). A median of 13 months (range 2–71) had elapsed between the birth of their infants and enrolment in the study. The South African PMTCT programme changed from the provision of single-dose NVP (sd-NVP) for PMTCT to the addition of AZT (initially from 28 weeks and later from 14 weeks of gestation) to sd-NVP. ART exposure in the mothers was therefore determined by the specific PMTCT regimen operative during their pregnancy, as well as the gestational age when they had presented to the antenatal clinic. Thirty-eight mothers had received sd-NVP and 11 had also received AZT (for between 7 and 192 days) as part of PMTCT. None of the mothers had any signs of active opportunistic infections at the time of study enrolment.

The median age of the infants in the mother-infant group was 13 months (range 2–71) with a male to female ratio of 1 : 1.47. The median age of the paediatric controls was 1.65 months (range 0.9–16.4). Thirty-four (74%) infants had received nevirapine after birth (for between 7 and 270 days) and seven infants had been on formula milk. The infants in the longitudinal group had a median age of 14.5 months (range 3–52) and a male to female ratio of 1 : 1. Twenty (90%) infants had received nevirapine after delivery and 3 had been on formula milk. All infants in the longitudinal group were started on HAART in the form of abacavir, lamivudine, and either ritonavir-boosted lopinavir (*n* = 19) or efavirenz (*n* = 3). Twelve infants started HAART at the time of the first study visit, 9 within one month of the first study visit, and one only after 3.8 months. One infant defaulted treatment in the first month and was reinitiated a month later. All infants were on cotrimoxazole prophylaxis.

The CD4^+^, CD8^+^, and VL results are shown in Table 1 for the entire cohort (*n* = 46), as well as for the subgroup (*n* = 33) for which flow cytometric analysis of CD4^+^ and CD8^+^ was available. The children had significantly higher CD4^+^ and CD8^+^ than the mothers [774 versus 330 and 1675 versus 740 cells/*μ*L, resp.], as well as significantly higher VL (980,000 versus 61,000 copies/mL). The CD4% was not, however, significantly different between the two groups (18% versus 22%), while the CD8% was marginally lower in children (43% versus 53%).

### 3.2. Immune Activation Profiles

These results are shown in Table 2. Relative to the adult control group, the HIV-infected mothers showed significant increases (*P* = 0.0242–*P* < 0.0001) in the concentrations of all the tested circulating biomarkers of immune activation. Similarly, HIV-infected children also showed significant increases (*P* < 0.0001) in the concentrations of all of the tested circulating biomarkers of immune activation relative to the control group, with the exception of MIG.

Fourteen of the forty-six (30.43%) children were diagnosed with TB. No significant differences in the concentrations of the various biomarkers were evident when comparing HIV^+^/TB^+^ coinfected children with the HIV^+^/TB^−^ group pre-HAART, except for *β*2M which was higher in the HIV^+^/TB^+^ group (*P* = 0.03) (data not shown).

### 3.3. Longitudinal Follow-Up of a Subgroup of the HIV-Infected Children

Twenty-two children on virologically suppressive HAART were followed up longitudinally from baseline to 14 months. These results are shown in Table 3. Significant increases in circulating CD4^+^ and significant decreases in VL were observed at 6 and 12 months after therapy. Relative to the baseline levels, children on virologically suppressive HAART showed decreases in *β*2M and MIG at 6 months and 12 months after therapy and decreases in sCD14 and IP-10 at 12 months after therapy. TGF-*β*1 remained elevated after therapy.

Although the above-mentioned biomarkers decreased after therapy, they remained elevated relative to the control group with regard to all of the biomarkers tested (*P* = 0.0494–*P* < 0.0001) with the exception of MIG, which remained significantly increased in the control group (*P* = 0.0143–*P* < 0.0001) (results not shown).

### 3.4. Effects of Maternal Smoking

Of the 46 HIV-infected mothers, 10 (21.7%) were smokers. The relationship between active smoking and the biomarker profile of this subgroup was assessed by multiple linear regression analysis that incorporated age, CD4^+^, CD8^+^, VL, and smoking in the mothers and infants with the inclusion of HAART in the mothers and feeding option and TB in the infants. While no significant associations were observed in the mothers, IP-10 was found to be significantly increased in smoke-exposed infants compared with infants without such exposure (*P* = 0.038) and was associated with CD8^+^ (*P* = 0.002). In addition, *β*2M was associated with infant age (*P* = 0.034), with younger infants displaying higher levels.

## 4. Discussion

This cohort of HIV-infected mothers recruited to the current study had low circulating CD4^+^ (median, 330 cells/*μ*L) in the setting of a high VL (median, 61000 copies/mL). As is well recognized, the children had significantly higher CD4^+^ than their mothers, but equivalent CD4%. The median VL was also significantly higher in the children, which is in agreement with previous reports documenting that HAART-naïve children tend to have higher VLs relative to those of adults [19, 20].

Relative to the healthy control group, all of the measured circulating biomarkers of immune activation were elevated in the group of HIV-infected mothers. These findings are in agreement with those of our previous study on circulating biomarker profiles of adult South Africans infected with HIV-1 subtype C on which the selection of the biomarkers evaluated in the current study was based [5] and are consistent with those of others focused on HIV-1 subtype B [4]. In our earlier study, *β*2M, IP-10, and MIG were found to decrease significantly after 6 months of virologically suppressive HAART and these biomarkers were considered to be possible predictors of successful therapy [5]. TGF-*β*1 and sCD14, on the other hand, remained elevated and were considered to be biomarkers of ongoing chronic immune activation [5], possibly related to microbial translocation [21].

In the case of the HAART-naïve, HIV-infected children, similar trends to those observed in their mothers were noted. The exception was MIG, the level of which was unexpectedly high in the control group of children. This latter finding is difficult to explain but may relate to a role for this chemokine in T-lymphocyte trafficking in the developing human immune system. In the subgroup of HIV-infected children monitored longitudinally, virologically suppressive HAART and recovery of CD4^+^ numbers were associated with significant decreases in the concentrations of circulating *β*2M and MIG at 6 and 12 months after therapy and sCD14 and IP-10 at 12 months after therapy. Although *β*2M and MIG may therefore complement measurement of VL in predicting responses to therapy in the setting of pediatric HIV infection, interpretation may be complicated by age-associated decreases in the circulating concentrations of these two biomarkers. For example, as demonstrated in this study and others, it is recognized that plasma concentrations of *β*2M are higher in neonates and infants up to one year of age, declining thereafter [22].

The late reduction in sCD14 is consistent with current awareness that significant effects of HAART on chronic immune activation occur slowly, which is in keeping with the observed lack of effect on TGF-*β*1 after 12 months of virologically suppressive HAART. This finding in relation to sCD14 is in agreement with an earlier South African study focused primarily on microbial translocation as a cause of persistent immune activation in HIV-infected infants aged ≤180 days [23].

With respect to smoking, it is well recognized that HIV-infected adults in North America and Europe not only have higher rates of smoking than their uninfected counterparts but also appear to be particularly vulnerable to the adverse health effects of smoking, even in the setting of virologically suppressive HAART [24–27]. Notwithstanding increased frequency and severity of pulmonary infections [28], HIV-infected smokers are particularly susceptible to the development of non-AIDS-defining conditions including cardiovascular disease, obstructive pulmonary disease, various types of cancer such as lung cancer, and associated early mortality [24–27]. Although the mechanisms underpinning the ominous interaction between HIV infection and smoking remain to be conclusively established, cumulative augmentation of chronic immune activation/immunosuppression seems likely [29].

In the African context, studies on tobacco use patterns in South Africa have revealed comparatively low rates of smoking in black African females of 7.5% and 4.6% in cohorts infected with HIV or coinfected with HIV/TB, respectively [30, 31]. Worryingly, however, a higher percentage of active smokers (21.75%) than those reported in the aforementioned studies was detected in the cohort of young African mothers recruited to the current study. Notwithstanding possible changes in the smoking habits of young South African females, these findings also raise concerns about the effect of parental smoking on chronic immune activation in young children in the setting of HIV infection, a seemingly underexplored public health issue.

In this context, the current study revealed that HIV-infected children exposed to maternal smoking showed significantly increased levels of circulating IP-10, which were significantly associated with CD8^+^. This finding is in keeping with the fact that IP-10 acts on activated T cells and macrophages [32], cell types which are increasingly viewed as important components of smoke-induced chronic inflammation [33]. Activated T cells release interferon-*γ* (IFN-*γ*), activating macrophages, which in turn release IP-10, MIG, and IFN-inducible T cell *α*-chemoattractant (I-TAC), which may promote chemotaxis of CD8^+^ via interaction with the CCR5 receptor, CCL5 [34]. In this context, it is noteworthy that levels of IP-10 are significantly increased in an in vitro cell coculture model of exposure of dendritic cells and bronchial epithelial cells to cigarette smoke condensate [35]. This finding is of potential significance since others have demonstrated that HIV-1 replication in monocyte-derived macrophages and peripheral blood lymphocytes is augmented in the presence of persistently elevated levels of IP-10, correlating with immunological treatment failure on HAART [32, 36]. On the other hand, no significant differences were observed between the immune activation profiles of active smokers and nonsmokers, possibly due to advanced disease with high VL levels or the extent of tobacco exposure.

The limitations of the current study include the following: (i) relatively small size of the study cohort; (ii) lack of perinatal data; (iii) the relatively short duration of HAART; and (iv) the age difference between the paediatric patients and controls. These are, however, off-set by several strengths of the study including documentation of (i) the existence of chronic immune activation in infants infected with HIV-1 subtype C; (ii) the apparent lack of utility of the measured biomarkers in monitoring the response to HAART in the paediatric setting, as opposed to adults; and (iii) the possible adverse effects of maternal smoking on chronic immune activation in HIV-infected infants. Although these apparent effects of maternal smoking must be considered as preliminary, the current study should serve as a template for future, more definitive studies, particularly in relation to parental smoking.

## Figures and Tables

**Table 1:** Demographic data of paired mothers and their children.

	Mothers [Median (range)] *n* = 46	Children [Median (range)] *n* = 46
Age	27 years (20–44)	13 months (2–71)
Circulating CD4^+^ T cell count (cells/*μ*L blood)	330^*∗*^ (115–744)	774 (22–2,856)
CD4^+^ T cells (%)	22^*∗*^ (7–37)	18 (0.9–45)
Circulating CD8^+^ T cell count (cells/*μ*L blood)	740^*∗*^ (265–2,942)	1,675^*∗∗*^ (181–6,301)
CD8^+^ T cells (%)	53^*∗*^ (32–77)	43^*∗∗*^ (18–67)
CD4/CD8 ratio	0.4^*∗*^ (0.1–1)	0.5^*∗∗*^ (0.1–2)
HIV-viral load (copies/mL blood)	61,000 (240–3,400,000)	980,000 (440–50,000,000)

^*∗*^Available data from 33 patients.

^*∗∗*^Available data from 32 patients.

**Table 2:** Comparison of the concentrations of circulating biomarkers of immune activation of mothers, their children, and their respective controls.

	Mothers baseline *n* = 46 [Median (range)]	Adult controls *n* = 20 [Median (range)]	*P* value	Children baseline *n* = 46 [Median (range)]	Paediatric controls *n* = 20 [Median (range)]	*P* value
sCD14 (ng/mL)	8,068 (0.00–19,431)	5,971 (853–8,220)	**<0.0011**	9,741 (481–20,448)	3,826 (0.0–21615)	**<0.0001**
*β*2M (*μ*g/mL)	2.84 (0.847–6.86)	1.360 (1.02–3.77)	**<0.0001**	4 (1.8–7.4)	2.135 (0.23–4)	**<0.0001**
hsCRP (*μ*g/mL)	1.8 (0.173–10.3)	0.8 (0.173–7.5)	**0.0242**	5 (0.2–144)	<0.173 (<0.173–8)	**<0.0001**
MIG (pg/mL)	2143 (595–19,734)	190.4 (84.9–2,932)	**<0.0001**	3,808 (1,058–13,818)	3,103 (158–16,563)	0.0779
IP10 (pg/mL)	6,866 (2,267–64,710)	1,075 (690–12,005)	**<0.0001**	7,171 (1,054–636,325)	21 (1.6–1,662)	**<0.0001**
TGF-*β*1 (pg/mL)	22,480 (52.8–33,685.5)	10,960.7 (3,157.6–31,806.7)	**0.0093**	12,627 (67–30,336)	11,374 (499–19,580)	**<0.0001**

sCD14 (soluble CD14), *β*2M (*β*2-microglobulin), hsCRP (high sensitivity CRP), MIG (monokine induced IFN*γ*), IP10 (IFN*γ*-inducible protein 10), and TGF-*β*1 (transforming growth factor beta 1).

**Table 3:** Longitudinal follow-up of a subgroup of the children.

	Baseline children Pre-HAART (*n* = 22) [Median (range)]	Children at 6 months Virologically suppressive HAART [Median (range)]	Children at 12 months Virologically suppressive HAART [Median (range)]
sCD14 (ng/mL)	10438 (960–18591)	7270 (889–15466)	6669^*∗*^ (734–13241)
*β*2M (*μ*g/mL)	4 (3–8)	2^*∗*^ (1.7–5)	2.5^*∗*^ (1.3–4.5)
hsCRP (*μ*g/mL)	4 (0.2–144)	1.3 (0.2–42)	1.6 (0.2–43)
MIG (pg/mL)	5312 (1250–11245)	1441^*∗*^ (611–11355)	813^*∗* *∗∗*^ (385–6081)
IP10 (pg/mL)	5559 (1054–64710)	6023 (1335–18749)	4614^*∗*^ (997–16679)
TGF-*β*1 (pg/mL)	14868 (5247–30336)	13419 (39–23940)	14117 (1021–18364)

CD4^+^ T cell count (cells/*μ*L)	854 (22–5915)	1724^*∗*^ (31–6401)	1731^*∗*^ (635–3828)
CD8^+^ T cell count (cells/*μ*L)	1471 (181–3128)	1819 (25–5984)	1785 (44–2792)
HIV-viral load (copies/mL blood)	2400000 (500–360000000)	25^*∗*^ (25–200)	25^*∗*^ (25–320)

HAART: highly active antiretroviral treatment.

^*∗*^For comparison with baseline values; *P* < 0.05.

^*∗∗*^For comparison with the 6 months' time-point; *P* < 0.05.

sCD14 (soluble CD14), *β*2M (*β*2-microglobulin), hsCRP (high sensitivity CRP), MIG (monokine induced IFN*γ*), IP10 (IFN*γ*-inducible protein 10), and TGF-*β*1 (transforming growth factor beta 1).
